# A Feat to Be Considered

**Published:** 1891-03-21

**Authors:** 


### THE DOCTOR'S ARMCHAIR.

### A FEAT TO BE CONSIDERED.

Lord Lonsdale has partly driven and partly ridden several different horses twenty miles in less than fifty-seven minutes, and has beaten the record. A certain class of persons will deride the feat as a mere jockey's performance. Jockey or no jockey, the thing could only have been done by a man of skill, pluck, endurance, and resource; and these are not endowments which any sensible person will think of despising. Brains are at a premium nowadays—or, rather, not brains, but mental circus tricks. How many facts can a man commit to memory, and reproduce at an examination-table? What new discovery can he make by gluing his eyes for ten or fifteen years to one of the minute peepholes of original research? These are the performances which the learning of the times prefers to skilled driving, football, cricket, or the thorough grounding in some useful art. Be not too sure, *Messieurs les pedagogues!* There is a good deal to be said on both sides.

It is plain that we must live in the world, and it is desirable that we should live with some sort of satisfaction. Life is not worth living unless the conditions be at least a little favourable. It is easy to imagine a concentration of evils upon any particular individual's head which would make death a happy event, and the grave a paradise. For example—A persistent asthma, coincident with an unremitting sciatica, accompanied by an incurable dyspepsia, and a "nagging" wife, will give us exactly the conjuncture we want to render life intolerable. This is only a sample of many similar sets of conditions which can easily be imagined, and many of which do actually meet in particular individuals and induce them to make their own "quietus, with a bare bodkin," or other available instrument. Self-destruction is by no means uncommon in England—in Germany and other Continental countries it is of decidedly frequent occurrence. Now, we may by familiarity learn to regard suicide as an event of no particular moment. But, whatever the event itself may be, the steps that lead to it are often a series of tragedies of the most painful and heartbreaking character.

Men like Lord Lonsdale never commit suicide; at least, they do not so long as riding and driving are the most exciting occupations of their lives. But why does a Lord Lonsdale not commit suicide? Because he enjoys his present life a great deal too much to think of prematurely exchanging it for another. Life was certainly given to us to be enjoyed. There can be no manner of doubt about that. There is no necessity to forget the facts of sorrow and pain, or the grievousness of almost all kinds of disciplinary processes. When all these things have been admitted, it is still a sound generalisation, which will stand the test of discussion, that man was intended to be happy, and can be happy if he only sets about it in the right way.

Happiness must have a physical basis. This is a fact which all sorts of wise and unwise people have agreed to forget. Men like Lord Lonsdale act upon this sound principle, although they are probably quite unconscious that they are doing so. The result is that they generally lead happy, simple, and wholesome lives. Perhaps there is nothing particularly great about the life of a man who is a mere amateur jockey. But there is no necessary reason why a man who is a first-rate jockey should be nothing else but a jockey. On the contrary, the presumption is that if he be liberally endowed with the qualities that go to make a good jockey, he is very likely to possess capacities which will make him successful in more intellectual and important pursuits.

But our point here is that in this world, and for men generally, happiness must necessarily have a physical basis. Without an adequate degree of physical health and well-being, very little experience which is worthy of being called by the name of happiness, is so much as possible. Are we, then, asserting that every bishop, every physician, every judge, and every rich and rotund man of the city should forthwith become a jockey? Not at all! But we do affirm that every bishop, and every physician, every judge, and every city man should try to find out the magic talisman which will lead him to the jockey's flow of spirits and the jockey's physical strength. Is such a thing possible? Yes! To every man who has a clear income of £500 a-year and upwards, and to most men who have not more than a fifth part of that income. It is a question of two things—intelligence and resolution. Among all the sorts of knowledge which are possible and desirable for men, the individual man must learn to choose and to properly value that which is most essential for his own happiness and well-being. But if it be true that happiness is impossible without a physical basis, then it is essential that all men who want to be happy should begin to acquire some knowledge of the physical basis of happiness, and of its needs and possibilities. This is exactly what nine persons in every ten positively decline to do.

How, then, may the man whom fate has not made a jockey set himself to work to acquire the jockey's health and spirits? The task is by no means easy. It takes rank with the conversion of Africans, and other such like problems. And the difficulties that lie in the way are precisely the difficulties which hinder African civilisation. Love of ease is the *vis inertiæ* which every average man finds it practically impossible to overcome. It is not entirely or even chiefly the love of physical ease: it is the indulgence of mental indolence that destroys health and deteriorates almost every man one meets. Nine-tenths of the persons who are in business for themselves, or who have secured the recognition of their individuality by means of their positive worth, could, if they were so minded, think out a plan of daily life which would give them the jockey's health and spirits; and they could manage to carry it out, at least approximately, in practice. But this would need a strict limitation of the hours of business, and a sufficient supply of really pure air every day, together with many other details. Eight hours a day should be the maximum period for any man to be shut up in a City office. During the greater part of that time he is inhaling poisoned air. Now poisoned air leads to definite degeneration of the bodily tissues. Therefore the other sixteen of the twenty-four daily hours should be spent in quite pure air; and certain steps should be taken to get rid of the injurious effects of the eight hours' inhalation of atmospheric poison, and also to rouse up the physiological energies to such a pitch as will produce high spirits and robust health.

But this involves living out of town, losing some opportunities of making headway in the world, and a whole host of inconveniences which cannot be contemplated by the eager business man without a shudder. Not a doubt of it! But, as he who would attain Heaven must pay the price of it, so must he who would attain health. The present writer would rather have Lord Lonsdale's energy, spirits, and pleasures, with £1,000 a-year, than Mr. Alderman Greenfat's, with his wheezy breath and waddling gait, with £20,000 a-year. Neither wealth, nor baronetcies, nor peerages, nor even that last and highest peak of the City man's mountain of human honour—a Lord Mayoralty—can for a single moment be compared with the brimming and effervescent happiness which come from robust health. The performance of Lord Lonsdale, and the whole question of physical and mental vigour, which is the result of physical exercise and a life in the open air, may properly be commended, first of all to every member of the medical profession, and then to every man and woman in the world who has the capacity for a little original thought.

